# Laryngeal Preservation Approaches: Considerations for New Selection Criteria Based on the DeLOS-II Trial

**DOI:** 10.3389/fonc.2019.00625

**Published:** 2019-07-10

**Authors:** Andreas Dietz, Susanne Wiegand, Thomas Kuhnt, Gunnar Wichmann

**Affiliations:** ^1^Clinic for Otorhinolaryngology, Head and Neck Surgery, University Hospital Leipzig, Leipzig, Germany; ^2^Department of Radiation Oncology, University Hospital Leipzig, Leipzig, Germany

**Keywords:** head and neck cancer, head and neck squamous cell carcinoma (HNSCC), laryngeal cancer, hypopharyngeal cancer, larynx organ preservation, induction chemotherapy, early response evaluation, decision-making

## Abstract

In the locoregional advanced group of larynx and hypopharyngeal squamous cell carcinomas (LHSCC), there are two kinds of patients: those who are candidates for functional larynx organ preservation (LP) by avoiding ablative surgery and those who are not. Currently, the distinction between them is depending on the patient's needs and desires, the experience and recommendation of the surgeon, the philosophy of the institution and others. The milestone VA trial established non-surgical LP in advanced LHSCC utilizing induction-chemotherapy (IC) with PF (cisplatin, P plus 5-fluorouracil, F) followed by irradiation (IC+RT) as appropriate alternative treatment to total laryngectomy (TL) already in the 1990s. Even thou the VA trial's findings were verified by the EORTC 24891 trial we have an ongoing debate about the best protocol balancing survival and laryngectomy-free survival (LFS) with acceptable late toxicity and good functional outcome. In advanced LHSCC without surgical options preserving the larynx, only IC+RT and primary concurrent chemo-radiotherapy (CRT) are accepted treatment options aiming to preserve a functional larynx. In the US, cisplatin-based CRT is still recommended as best protocol to achieve cure of the disease and LP. But current views on long term survival with functional organ preservation and still high failure rates are addressing the need of better selection of patients which will be discussed as follows taking the current debate in literature and in particular the recently published data of the DeLOS-II trial in consideration.

## Functional Organ Preservation in Larynx Cancer: A Continuing Debate

The authors of the worldwide most visible LP-trial, the phase III, 3 arms RTOG 91–11 trial, essentially adhere rigidly to their earlier recommendations also in 2013.Unchanged assumed from the 2006 publication augmenting the superiority of CRT over alternative non-surgical approaches in terms of larynx preservation they wrote: “CRT offers a significantly higher chance of larynx preservation than RT alone or induction chemotherapy followed by RT, albeit at the cost of higher acute in-field toxicities and without improvement in overall survival (quality of evidence: strong; strength of recommendation: high) (1). The RTOG 91–11 study and respective publications are lacking current state-of-the-art functional follow-up reporting. Larynx function in its different multiple dimensions is essential for the highly complex interplay of swallowing, speaking, breathing, and others like abdominal tension and bowel movement. To address all aspects, modern LP-trials should stress dysphagia follow up screening, more specific voice and late toxicity assessment, and health related quality of life issues. To be provocative, in RTOG-91-11 the relevant definition of functional organ preservation is not met, and RTOG assessed organ preservation only as “organ in place.” Addressing questions concerning swallowing dysfunction, such as “intake of only sieved food preparations,” resulted in a worse outcome in the simultaneous CRT group (20.5 vs. 13.5% in the induction group). Moreover, the CRT arm showed lower compliance and a much higher rate of “late death unrelated to cancer” (36% in the CRT arm vs. 18% in both comparator arms, RT or IC+RT). Giving more attention to these functional aspects, data of the RTOG 91–11 strongly suggest that induction seems to be the better option for larynx organ preservation due to several reasons.

**Figure 1:**
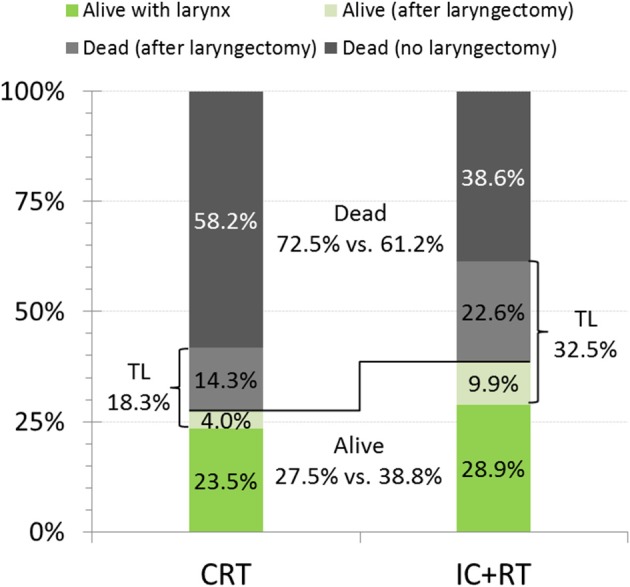
Ten-years outcome data of patients treated with concomitant cisplatin radiotherapy (CRT) vs. induction-chemotherapy with cisplatin and 5-fluorouracil followed by radiotherapy (IC+RT) in the Radiation Therapy Oncology Group (RTOG) phase III randomized clinical trial RTOG 91–11 demonstrates superior long-term survival and survival with larynx despite increased frequency of total laryngectomies (TL). Modified representation of data from Forastiere et al. (1) according to Licitra et al. (2).

Based on a recently published reanalysis of the RTOG 91–11 (2), Licitra et al. stated that the results of the trial would not show any clinical superiority over IC+RT in the context of LP. Analyses using the cumulative incidence method mislead the interpretation in the concomitant arm associated with larynx preservation in a delusive optimistic direction, as there was a higher mortality in the concomitant arm (CRT) that reduced the cumulative incidence of TL in the CRT arm and deaths after TL are not reported for both arms, CRT and IC+RT. This would depict the pretended superiority in LP with CRT over IC+RT (hazard ratio, 0.58; 95% CI, 0.37–0.89; *p* = 0.005) and result in a completely different picture (Figure 1). The proportion of patients alive at 10 years was definitively higher with IC+RT over CRT: overall survival was 38.8 vs. 27.5%, survival with larynx was 28.9 vs. 23.5%, and survival without larynx was 9.9 vs. 4%, respectively (Figure 1).

Consistent to their upper mentioned analysis Licitra et al. summarized, the results of RTOG 91–11 fail to provide any support for the superiority of CRT. This suggests that sequential treatment by applying IC+RT is more effective and achieves an substantial increase in the proportion of long-term survivors with and without larynx (Figure 1) (2).

Nevertheless, the major disadvantage of LP by CRT or IC+RT is the not so seldom tumor persistence after completed per-protocol treatment or early relapse after curative treatment requiring salvage surgery. Head and neck surgeons worldwide share the observation that salvage surgery showed to be suitable after CRT in principle but might cause major complications and not being feasible in a relevant number of patients. Salvage surgery and in particular performed after a longer time since irradiation is prone to complications (3–8). Moreover, salvage surgery is associated with a significantly prolonged time until wound healing is achieved, a higher probability of fistula, requirement of flap surgery for reconstruction and wound closure resulting in longer hospitalization and reduced quality of life (3, 4, 9). Leemans et al. demonstrated that in nearly 50% of cases with resectable disease going in line with acceptable general health condition allowing safe surgery before CRT, salvage surgery was not feasible after definitive CRT due to increased comorbidity, onset of new metastases, lacking motivation of patients and other reasons (5, 10).

By considering all the above mentioned factors, Lefebvre and Ang (11) defined the major goal for future larynx organ preservation trials as laryngoesophageal dysfunction-free survival. Moreover, they recommended a restriction of LP trials and the indication for LP utilizing IC+RT or CRT to big larynx-T3-tumors in general, indicating that T4a might end up with lower functional preservation rates. However, the analysis of T4-larynx cancer data from high-volume centers (e.g., the MD Anderson Cancer Center) demonstrated unacceptable functional outcome after CRT and significantly better local recurrent-free survival after TL in this patient group. Patients treated with initial TL had more distant metastases and lower overall survival rate (*p* = 0.7). For selected T4 patients with smaller tumors and more limited disease who were expected to have a low probability of locoregional failure achieved with the non-surgical therapy good locoregional control combined with preserved airway, and also adequate and safe swallowing could be reserved (12). Furthermore, Grover et al. (13) presented retrospective National Cancer Data Base data from 969 patients with T4a-larynx carcinoma (M0, treated between 2003 and 2006). Compared to the 64% of T4 larynx-cancer patients receiving primary CRT those 36% treated with TL with or without adjuvant radio/chemotherapy had significantly better 5-year overall survival (*p* = 0.001). Therefore, the MD Anderson Cancer Center recommends in line with the Lefebvre and Ang recommendation to restrict the indication for non-surgical treatment regimens to T3 and small selected T4a cancers. Additionally, same sounding findings were described in a retrospective single institution observation from Netherlands showing that non-surgical organ preservation approach is only survival equivalent in T3 but not T4a cancers compared to TL with 42 vs. 48% after 5 years (14).

Furthermore, it should be discussed whether the limited view on pure macroscopic pattern of tumor extent are adequate to balance the optimal different treatment for the individual patient despite availability of individual biologic and highly dynamic early therapy response specifics which are predominantly ignored in the current debate. Most early identification of patients who are not likely to benefit from non-surgical treatment should be mandatory. Anyway, PF-based IC+RT causes less severe late toxicity, increases the LFS due to a lower rate of death unrelated to cancer and therefore in the long run is superior to CRT (1). As IC strongly improves patient selection for adequate treatment, IC is recommended as relevant part of multimodal LP-protocols. As new therapeutics including targeted therapies and their combination with chemotherapies and of chemotherapy with immune-checkpoint inhibitors are emerging (15), further development of LP by IC+RT is still under consideration. IC with TPF, the combination of docetaxel (T) with PF, is superior to PF as nearly identical demonstrated in the TAX 323 and TAX 324 trials (16–18). Furthermore, the GORTEC 2000-01 trial specifically designed for LP demonstrated acceptable feasibility, efficacy and superiority of TPF compared to PF (19). The first trial of the German Organ Preservation Study Group (DeLOS-I) demonstrated the feasibility of TP-based (carboplatin plus paclitaxel) IC+RT in LP with encouraging low rates of late dysphagia after 3 years (20).

## DeLOS-II Trial

Nearly the same time of the presentation of TAX 323 and TAX 324 data new publications introduced the cetuximab as a treatment for LHSCC as this humanized antibody by binding the epidermal growth factor-receptor (EGFR) increased efficacy of radiotherapy (21, 22). The randomized phase II DeLOS-II-trial (NCT00508664) was designed as a study to the use of TPF-chemotherapy followed by radiotherapy with or without cetuximab (arm A TPF; arm B TPF ± E) in the primary therapy of only by TL operable carcinomas of the larynx/hypopharynx. DeLOS-II should answer the questions if (1) adding cetuximab would be able to further increase the already high response rates to TPF, and (2) if such potentially higher response rates translate into higher larynx preservation rates (23, 24). DeLOS-II introduced first in literature the empirical cut-off of ≥30% tumor surface shrinkage, a reduction estimated after the first cycle IC (short induction) based on endoscopic re-evaluation by the surgeon (23). This simple but effective early evaluation method with an endoscope was empirically developed since any imaging method after one cycle IC does not show reliable differences analyzing these low volume and superficial spreading tumors. This method belongs to the routine diagnostic procedure of any ENT- or Head & Neck specialist and therefore gives a high degree of certainty due to direct and measurable feedback during the first weeks for the treating team. Endoscopic early evaluation was used for discrimination between early responders after one cycle IC receiving further two cycles IC followed by RT (≥30% tumor surface shrinkage), and non-responders undergoing safe TL before relevant tissue damaging further treatment per-protocol (24).

Despite being accompanied by an elevated frequency in adverse events, the IC with TPF/TP plus cetuximab according to the DeLOS-II data was feasible but showed no superiority to IC with TPF/TP regarding LFS and OS at 24 months. Both early response and 24 months LFS compared very well to previous larynx organ preservation trials and recommended effective treatment selection and stratification by ≥30% endoscopically estimated tumor surface shrinkage. Until 24 months, salvage TL was not carried out in 115 of 173 patients. In 51 patients, salvage TL was indicated, feasible and done. Per-protocol, salvage LE was planned for 37 poor-responders (<30% endoscopically estimated tumor surface shrinkage in week 4; 21.4%). In spite of intensive counseling, only 57.4% of poor-responders agreed to the operation (24).

As the DeLOS-II protocol introduced the early endoscopic re-evaluation and defined eligibility criteria to select salvage TL for non-responders after only 3 weeks, the question arose how reliable endoscopic re-evaluation reflects the response measured e.g., by volumetric and other assessments.

Moreover, there remained so far unresolved questions regarding the best selection criteria for matching the right patient and the optimum treatment to achieve longtime LFS without harming oncological safety. There is still an ongoing search for markers with the highest discriminative power facilitating decision-making how to treat a patient achieving both aims. Potential selection criteria include anatomical as well as pathological characteristics of the malignancy (in particular T and N categories) and have to consider especially those linked to poorer local control rates, e.g., large tumor size and localization of the primary tumor in the hypopharynx (25, 26). However, molecular features (27–29), but also the volume of the primary tumor, tumor penetration of the cartilage, local neck metastases, and high PET detected metabolic rate of the tumor (30) are well-known critical factors requiring consideration in the decision-making process.

We designed a study embedded in DeLOS-II with 52 patients to find out if (a) an appropriate decision-making process is possible by using only clinical information plus endoscopic assessment of the tumor's response to first cycle IC by applying the ≥30% criterion, or (b) is best facilitated by computed tomography (CT)-based volumetry, or (c) ^18^F-FDG-PET/CT, or (d) essentially needs the combination of all assessments. By clarifying these questions we are now able to propose the LFS-score, a score derived from independent predictors out of a multivariate Cox regression model that by combining the essential information from clinical and radiological assessments has the potential to improve selection of patients for non-surgical LP without compromised survival by IC+RT (31).

## Laryngectomy Free Survival Predictive Score for Non-Surgical Organ Preservation: LFS-Score

**Figure 2:**
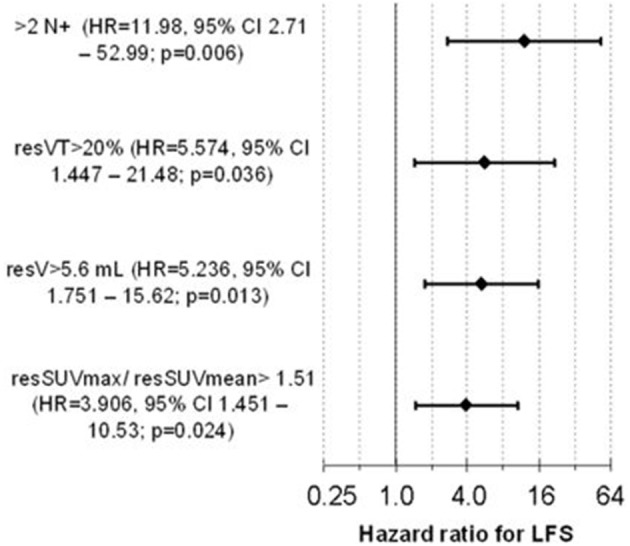
The Cox proportional hazard model for laryngectomy-free survival (LFS) developed in responders (p = 6.28 × 10–4) contains 4 independent significant covariates and predicts the LFS, overall (OS), and tumor- specific survival (TSS) of responders Forrest plot for the four covariates in the multivariate Cox model (1) suspect positive neck nodes (N+) ≤2 vs. >2, (2) residual volume of the primary tumor resVT ≤20 vs. >20%, (3) residual total tumor volume in CT-based volumetry resV≤5.6 vs. >5.6 ml, and (4) the ratio of the residual standard-uptake value maximum and the residual standard-uptake value mean resSUVmax/resSUVmean≤1.51 vs. >1.51. Fifty two consecutive patients with advanced laryngeal and hypopharyngeal carcinoma in the phase II larynx preservation trial DeLOS-11 treated in the University Hospital Leipzig (31); permission for reuse kindly provided by Elsevier.

**Figure 3:**
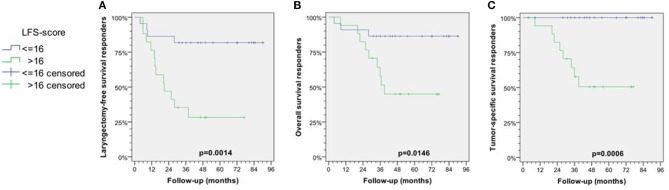
Kaplan-Meier curves for laryngectomy-free survival (LFS; **A**), overall (OS; **B**), and tumor-specific survival (TSS; **C**) among responders stratified according their LFS score ≤16 vs. >16 are shown together with *p*-values from log-rank tests. Modified reproduction from Wichmann et al. (31); permission for reuse kindly provided by Elsevier.

We used the HR of the 4 independent predictors as upper mentioned to develop an LFS-score to predict the individual suitability for larynx preservation applying the DeLOS-II protocol. Absence of a negative predictor scored 0, while either its presence or missing information scored with their rounded HR, 12, 6, 5, 4 for ≥3 N+, resVT>20%, resV>5.6 mL, and resSUVmax/resSUVmean>1.51, respectively (Figure 2). We identified 16.5 as optimum cut-off for the LFS-score and proved the sum of LFS-score>16 (potential LFS-score max = 27, in responders observed LFS-score mean = 13.7, range 0–27) being predictive for better outcome after TL. In our cohort of DeLOS-II patients, responders with LFS-score <16 had significantly improved LFS, OS, and TSS (Figure 3) (31).

In early responders, 15/16 patients with salvage TL or tumor-related death had a LFS-score>16 and therefore were correctly predicted. The positive predictive value (PPV, specificity) for being a candidate for TL was 93.8%. Four patients with LFS-score ≤ 16 survived but lost their larynx (one survivor lost his larynx after 9 months, three died without relation to cancer after 4, 9, and 28 months). 18/23 patients with successful LP were correctly identified by LFS-score ≤ 16 (NPV vs. TL = 78.3%). Correspondingly, 5/23 (21.7%) of patients with LP would have the recommendation for TL according to their LFS-score > 16. Applying the LFS-score > 16 as criterion to non-responders also recommends TL as adequate treatmnent (31).

LP vs. salvage TL in responders was rather dependent on LFS-score ≤ 16 (*p* = 0.001) than T category (T2/T3 vs. T4a; *p* = 0.173) or localization (larynx vs. hypopharynx; *p* = 0.584). Obviously this score is the most important factor for superior outcome in responders regarding LFS, OS and TSS (all *p* < 0.05). A LFS-score > 16 was detected in 1/4 (25%) T2, 9/22 (40.9%) T3, and 8/13 (61.5%) T4a LHSCC; 0/6 N0, 1/4 (25%) N1, 7/15 (46.7%) N2b, 10/13 (76.9%) N2c, and 0/1 N3 LHSCC had a LFS-score > 16. LFS of responders with LFS-score ≤ 16 was not different in T4a vs. others or larynx vs. hypopharynx carcinoma. A “deep response” (endoscopic tumor surface shrinkage >70% at about 3 weeks after only the first cycle IC) was found to be significantly correlated with LFS-score <16 (*p* = 0.016) and translated into a superior LFS (*p* = 0.047) (31).

We provide evidence that the decision for LP or TL in advanced LHSCC should integrate clinical information about shrinkage of the primary tumor (at least 30% surface shrinkage as estimated by the treating head and neck surgeon) and additionally the calculation of the LFS-score based on the number of suspect positive (N+) neck nodes and the response-assessment after IC-1 with ^18^F-FDG-PET/CT and CT-based volumetry to provide the required measures. The internally (by bootstrapping applying 1,000 iterations) validated Cox model and the newly developed LFS-score can facilitate proper decision-making. Their use may improve OS by reducing the probability of relapse and salvage TL, those risk factors being with the highest impact on survival strongly correlating with dying from cancer (*p* = 0.001, *p* = 0.015) (31). Locoregional failure and losing the larynx was a main reason to die also in other IC+RT trials (32). Therefore, and because of uncompromised TSS of early laryngectomized non-responders, a reliable prediction of the chance for successful LP in early responders is most critical for TSS. As the LFS-score allows to distinguish responders with LP and uncompromised TSS and LFS (LFS-score ≤ 16) from those at risk (LFS-score > 16), the decision-making regarding IC+RT vs. TL in responders (especially those with ETSS between 30 and 70%) may benefit from using the LFS-score.

The data distribution in the response-parameters assessed in Wichmann et al. (31) show a higher variance in particular response parameters especially in non-responders; they more often had responses in one given parameter not correlating with other response parameters. Therefore, univariate significant response-parameters do not correlate well with other univariate significant parameters from other assessments. Consequently, a non-critical alternate use of any response parameter instead of another seems to be misleading. IC+RT has the potential to achieve LP even in LHSCC stage IV, T4a, and N2 or N3, whenever a strong response to IC-1 is observed and the LFS-score combining the essential information from clinical and radiological assessments is ≤16 (Figure 3).

Due to cost and availability of PET/CT scanners the LFS-score analysis in Wichmann et al. (31) was based on patients only treated in Leipzig (52/173 randomized DeLOS-II patients). However, this is linked to the benefits of mono-centric studies: All processes were optimized, only two well-experienced ENT surgeons assessed eligibility of the patient for the trial and the tumor before treatment and its response to IC-1 endoscopically; all PET/CT-scans were performed using one scanner by the same radiologist; the team treating the patients was unique and well-trained. Advantageously, all results should have low inter-observer variability. It is of importance that the LFS-score appears to be able to serve as an indicator for significant outcome differences in LHSCC showing a huge heterogeneity in clinical characteristics and many other response-associated parameters (31).

## Conclusion and Outlook

It is very likely that the now improved patient selection processes, refinements in radiotherapy technique, and new drug combinations now including targeted and immune-checkpoint inhibitors will provide different outcomes from those obtained in RTOG 91–11 patients treated more than 20 years ago (1, 2) as we observed in the DeLOS-II trial remarkable improvements compared to earlier data. As outlined in our earlier paper, good decision making requires familiarity with decision-relevant factors and recognition of the values relevant to weighing the pros and cons of the alternatives, i.e., in advanced LHSCC balancing functional larynx preservation and oncologic safety (31). The LFS-score probably is able to facilitate the decision-making processes in IC+RT by proving additional indicators defining patients with high probability to survive without relapse and functional larynx preservation. As we know from several lines of evidence from DeLOS-II and in particular our subgroup of 52 patients, the total laryngectomy should be recommended as early as possible and should be done before radiotherapy as this effectively prevents further growth of the tumor often leading to unsuitability of salvage surgery after full protocol intake, severe salvage complications, distant metastasis and tumor-related death. Following the DeLOS-II-observations, hypopharyngeal and T4a LHSCC might equally show excellent results after larynx organ preservations treatment, provided their individual early response behavior i.e., LFS-score gives advice. Doing the assessments before starting IC and 3 weeks after IC-1 allows to combine information from endoscopic assessment, ^18^F-FDG-PET/CT, and CT-based volumetry appears to be able to prevent cancer-related death and thus worth the additional effort. However, in a shared decision-making process, where clinicians are asked to transmit information to individual patients regarding the probabilities of IC-RT, CRT and TL for being alive with or without larynx, competing causes for death like cardiovascular and chronic obstructive pulmonary disease, and second primary (lung) cancer require also to be taken into consideration (33–40). Due to the often high exposure to risk factors like smoking and alcohol and the high degree of comorbidities in a patient, laryngectomy in advanced LHSCC is often less incriminating than any primary IC+RT or CRT (33–40).

Putting the take home message in a nut shell, individual decision making based on early response evaluation after one cycle of IC for better selection of good larynx preservation candidates is the key.

## Author Contributions

All authors listed have made a substantial, direct and intellectual contribution to the work, and approved it for publication.

### Conflict of Interest Statement

AD and GW: research grant by Merck Serono. AD: honorarium for advisory boards from Merck Serono. The remaining authors declare that the research was conducted in the absence of any commercial or financial relationships that could be construed as a potential conflict of interest.
